# Supplementary figures and images for: Low affinity binding sites in an activating CRM mediate negative autoregulation of the Drosophila Hox gene Ultrabithorax
Source: PLoS Genet. 2019 Oct 7;15(10):e1008444. doi: 10.1371/journal.pgen.1008444 (PMC6797233; doi:10.1371/journal.pgen.1008444)

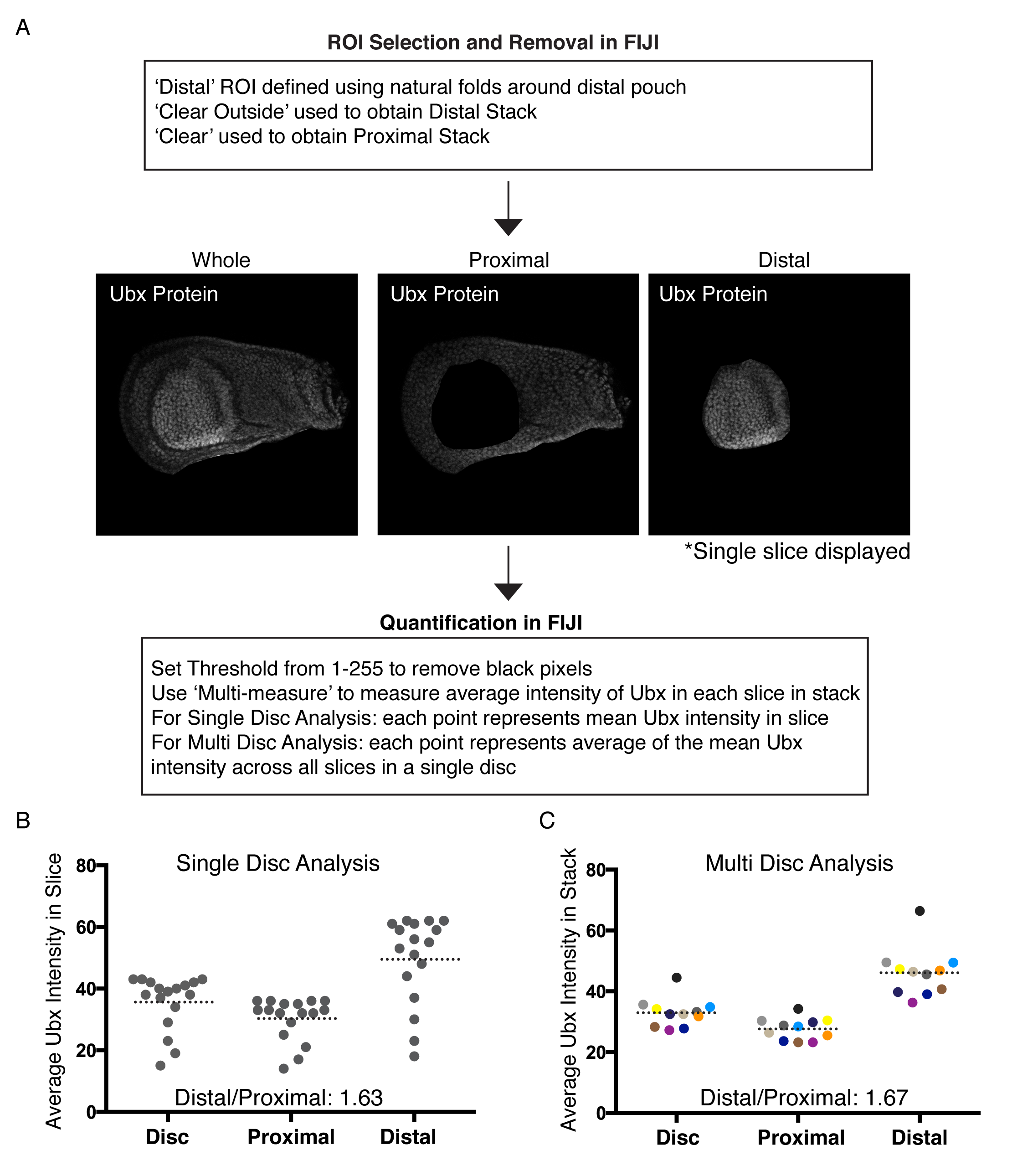

Supplement: S1 Fig — (A) Analysis pipeline conducted within FIJI is shown. Selection of whole disc region of interest (ROI), Distal ROI, and Proximal ROI is followed by the measurement of the average intensity of Ubx within that ROI for each slice in the stack. Black pixels are removed from the analysis to prevent skewing of the average from differences in sizes of the ROI. (B) (Left) An analysis of a single haltere disc is shown. Each dot represents the average intensity for each slice in the stack in the whole disc (“Disc”), the proximal ROI, and the distal ROI. Dotted line represents the mean for each compartment; reported distal:proximal ratio is the distal mean/proximal mean. (C) As shown in Fig 1. The mean average Ubx intensity for each compartment from the single disc analysis is reported as a single point in the multi-disc analysis. Individual discs are color-coded such that the compartment-specific averages for each disc can be compared to one another. Dotted line is the mean average intensity for each compartment. (TIF) [file pgen.1008444.s001.tif]

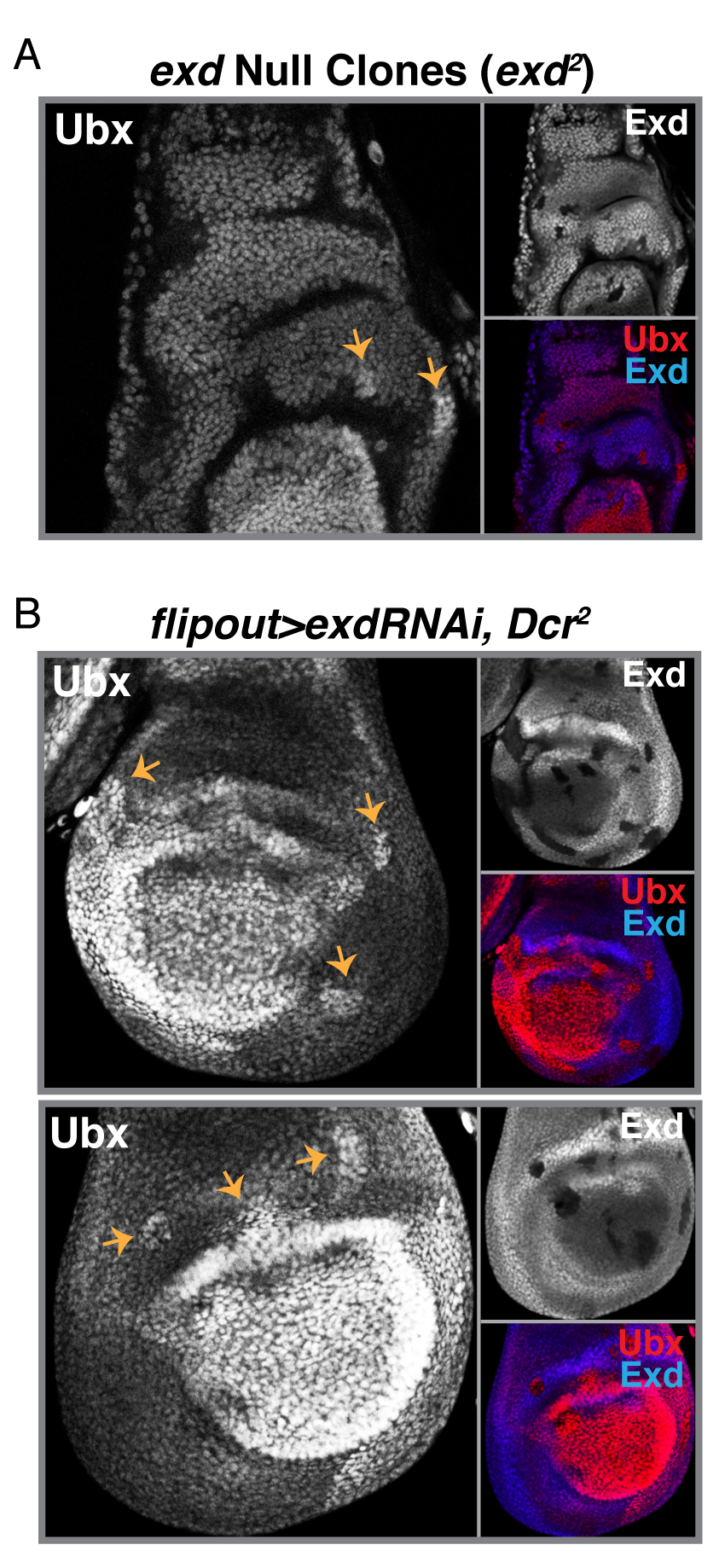

Supplement: S2 Fig — (A) Ubx Immunostain in haltere discs in which exd null clones (exd2) have been induced. An Exd immunostain, in addition to a merge of Ubx/Exd, is shown. Clones are marked by a yellow arrow. (B) Ubx immunostain in two haltere discs in which exdRNAi clones have been induced in the background of a Dcr2 mutant. An Exd immunostain, in addition to a merge of Ubx/Exd, is shown. Clones are marked by a yellow arrow. (TIF) [file pgen.1008444.s002.tif]

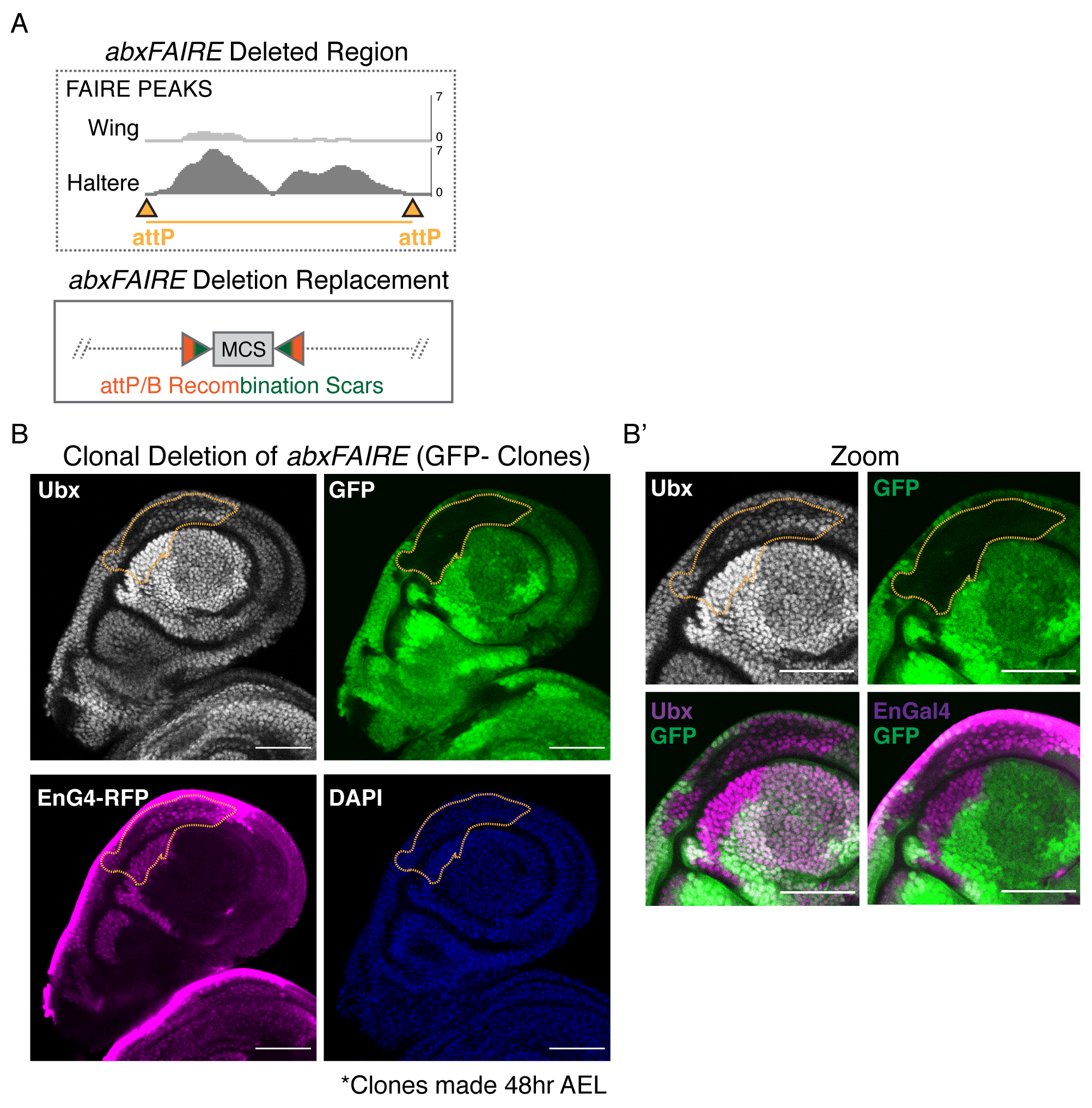

Supplement: S3 Fig — (A) (Top) A schematic of the abxFAIRE region targeted and deleted. FAIRE accessibility peaks [28] are shown in the wing and haltere. (Bottom) The ΔabxFAIRE allele was generated by replacing the abxFAIRE sequence with a minimal cloning site (MCS) sequence using PhiC31-based RMCE (see methods). Through this method, scars are left on either side of the replacement. (B) Mitotic clones homozygous for ΔabxFAIRE (GFP-) were induced at 48hr after egg-laying (AEL). Clones in the posterior compartment (EnGal4-RFP+) do not show a defect in Ubx expression. Clone is outlined in yellow. (B’) A zoomed in image of the clone from B. Merges of Ubx/GFP and EnGal4-RFP/GFP are shown. Scale bars are 50 micron in size. (TIF) [file pgen.1008444.s003.tif]

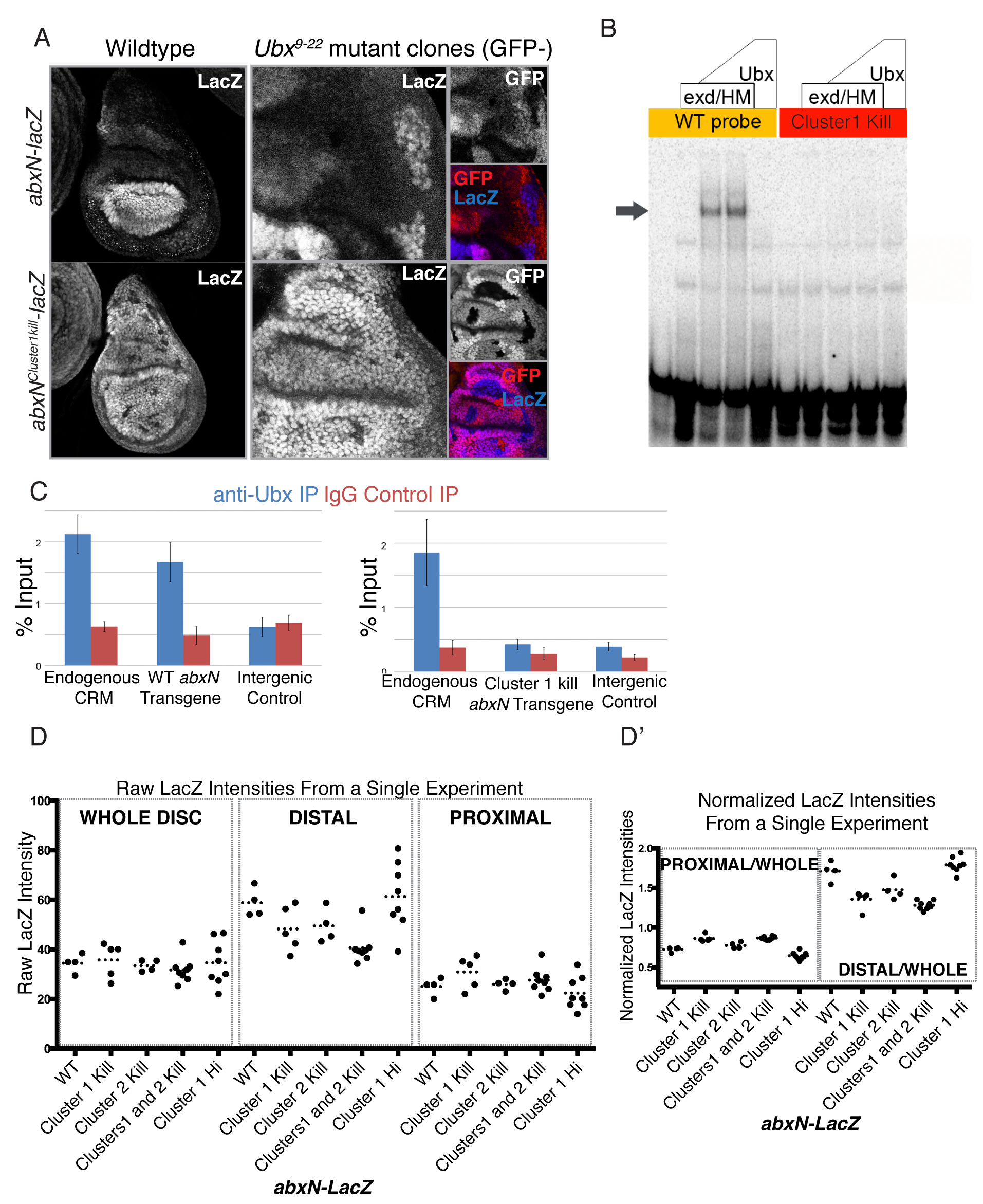

Supplement: S4 Fig — (A) (Left) LacZ immunostain of haltere discs containing either an abxN-lacZWT transgene or an abxN-lacZCluster1-Kill transgene. (Right) LacZ immunostain of discs of wildtype and mutant abxN-lacZ genotype upon induction of Ubx null clones (Ubx9-22). A GFP immunostain, in addition to a merge of GFP/LacZ, is shown. Clones are GFP-negative. (B) EMSA assay of Ubx-Exd-HthHM in vitro binding to a probe containing the Cluster 1 binding sites (left) and the probe mutated to abrogate the Cluster 1 binding sites (right). Gray arrow points to the shifted trimer band. (C) Chromatin Immunoprecipitation (ChIP-qPCR) was performed on haltere discs from transgenic flies containing either the abxN-lacZWT reporter (left) or the abxN-lacZCluster1-Kill reporter (right). % Input for an anti-Ubx IP and an IgG Isotype control IP are shown for three genomic regions: (1) the endogenous abx CRM, (2) the transgenic CRM, and (3) an intergenic region on chromosome 2 that serves as a negative control. Averages and standard deviation from three independent IPs are reported. (D) Quantification of raw LacZ intensities in discs from a single experiment out of a total of three, which were reported together in Fig 4C and 4C’. Values are reported for the average LacZ intensity of the whole disc, the distal compartment, and the proximal compartment. Each dot represents a single disc, and the dotted line signifies the mean value. While the trend in the data is representative of that in Fig 4C, none of the differences here are statistically significant (ANOVA, cutoff .05), likely because of the large amount of variability in these measurements and small sample size of a single experiment. (D’) Quantification of normalized LacZ intensities from D. Proximal and Distal LacZ average intensities for each disc are divided by the average intensity of the whole disc. This quantification provides a measurement of the contribution of proximal and distal LacZ levels to the average of the whole disc. An inverse relatio [file pgen.1008444.s004.tif]

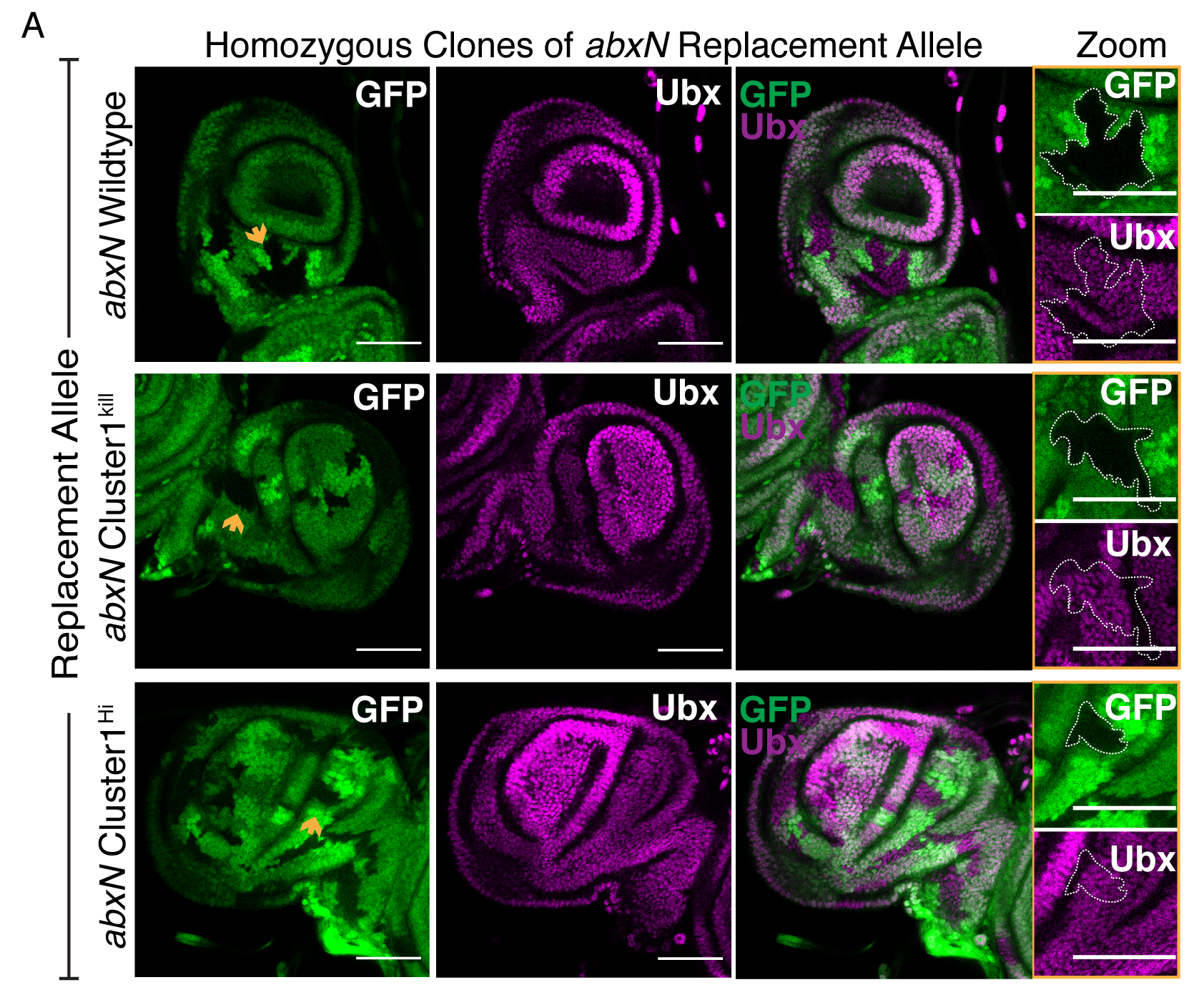

Supplement: S5 Fig — (A) GFP and Ubx immunostains in haltere discs in which clones homozygous for abxN replacement alleles were induced 48hr AEL. Clones are GFP-negative and denoted with a yellow arrow. Zoomed images of single clones (outlined) are shown to the right. All scale bars shown are 50 micron in size. (TIF) [file pgen.1008444.s005.tif]

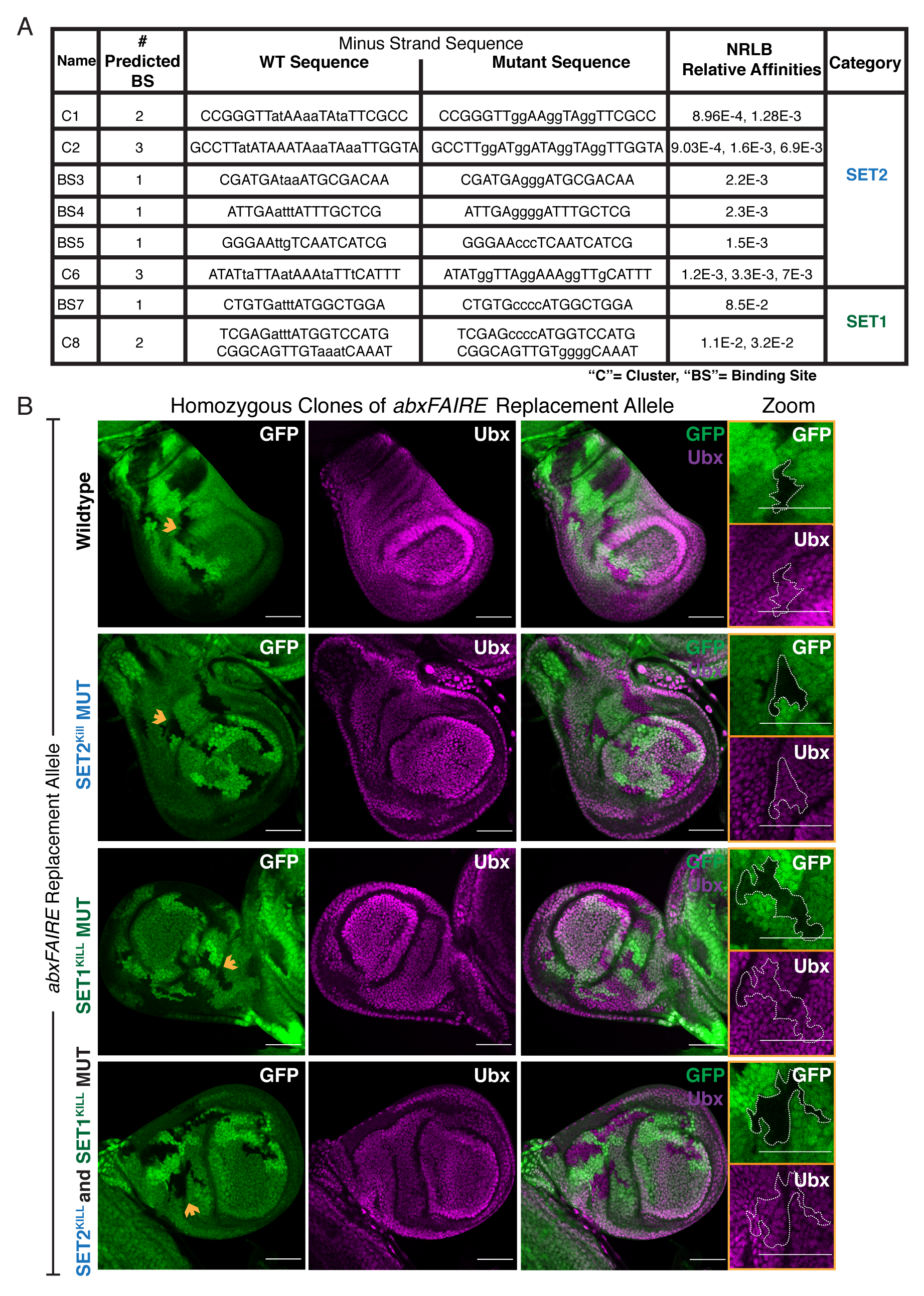

Supplement: S6 Fig — (A) Table of NRLB-predicted Ubx/Exd binding sites in abxFAIRE that have been mutated. Many predicted sites fall within clusters so we report the number of binding sites within each cluster. Wildtype and mutated sequences are shown. Lowercase levels are bases that were mutated. Relative affinities and the category (Set1, Set2) for each binding site or cluster are given. (B) GFP and Ubx immunostains in haltere discs in which clones homozygous for abxFAIRE replacement alleles were induced 48hr AEL. Clones are GFP-negative and denoted with a yellow arrow. Zoomed images of single clones (outlined) are shown to the right. All scale bars shown are 50 micron in size. (TIF) [file pgen.1008444.s006.tif]

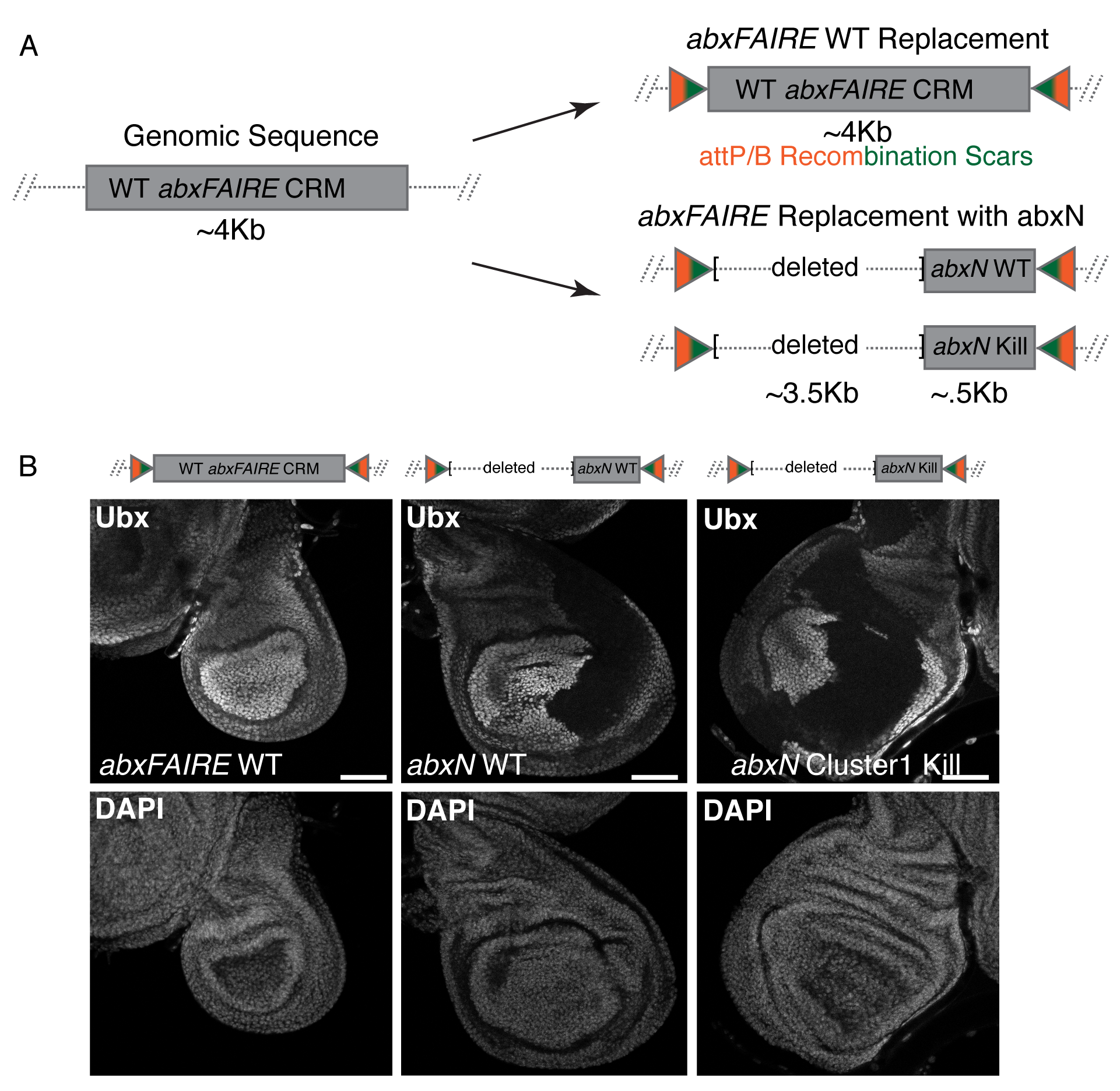

Supplement: S7 Fig — (A) Schematic of abxFAIRE replacements tested in B. The Targeted Region4kb replacement platform was used to generate either a wildtype abxFAIRE (+scars) allele or an allele with only the ~500 bp abxN wildtype or Cluster 1kill sequence, deleting the remaining ~3.5 kb abxFAIRE sequence. (B) Ubx immunostain in haltere discs homozygous for the specified abxFAIRE replacement alleles. A DAPI nuclear stain is shown for each. All scale bars shown are 50 micron in size. (TIF) [file pgen.1008444.s007.tif]

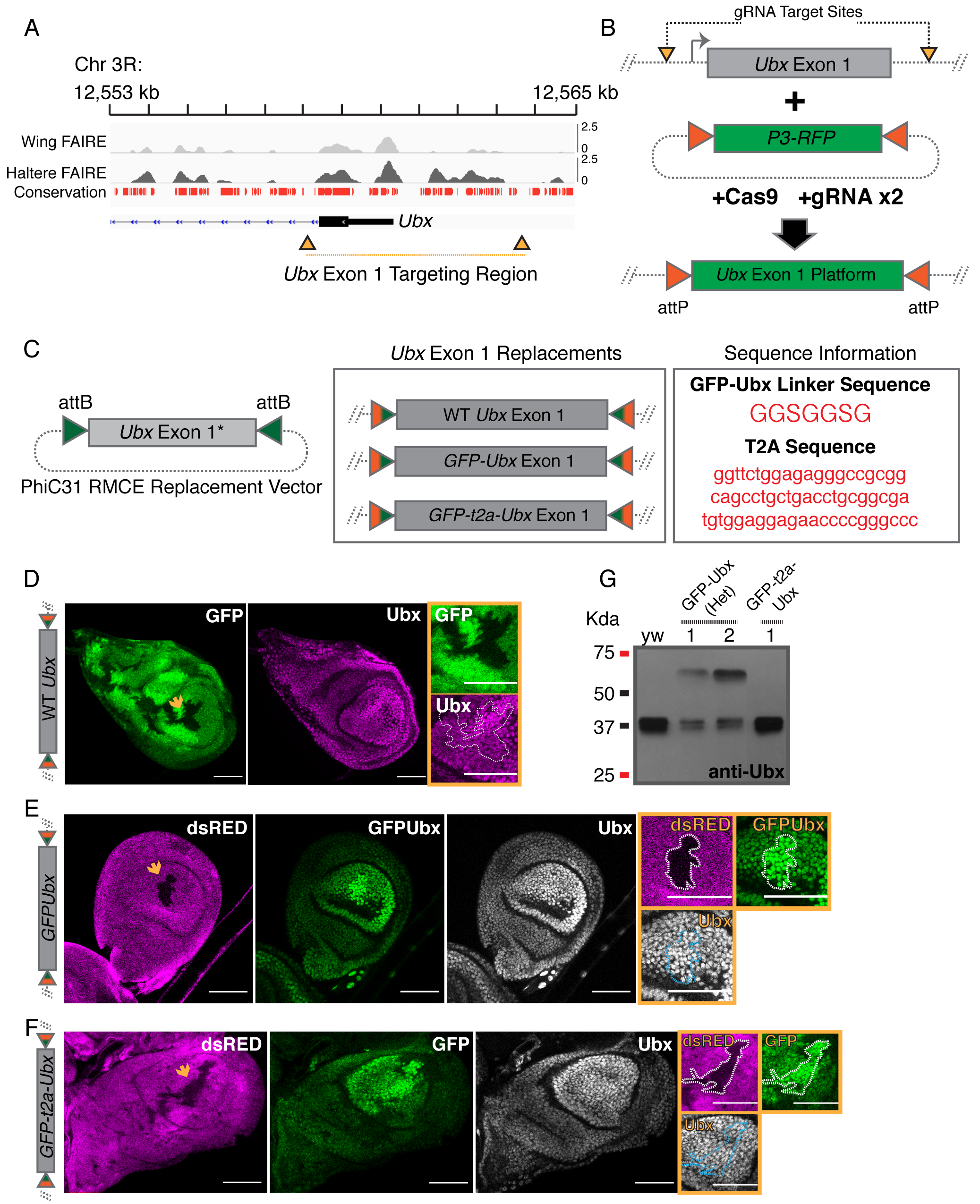

Supplement: S8 Fig — (A) Genome browser screenshot around Ubx exon 1. FAIRE accessibility peaks in the wing and haltere [28] are shown for reference. Conservation track was downloaded from UCSC. No bars denote a lack of evolutionary conservation with twelve other Drosophila species, mosquito, honeybee and red flour beetle. Yellow triangles denote Cas9/gRNA target sites; and the yellow bar denotes the region replaced. (B) Schematic of a two-step Ubx exon 1 replacement strategy. gRNA sites were chosen in non-conserved regions surrounding Ubx exon 1. A dual-gRNA expressing plasmid was injected along with a donor cassette containing an attP-flanked fluorescent selection marker (P3-RFP) into nanos-Cas9 flies. The resulting “Ubx Exon 1 Replacement Platform” serves as a means to insert modified versions of Ubx using PhiC31-based RMCE. (C) (Left) Schematic of RMCE replacement cassette. (Right) Schematic of replacement alleles generated and sequence information for the linker used in the GFP-Ubx fusion (amino acids) and the T2A sequence used (DNA). (D) GFP native fluorescence and a Ubx immunostain in haltere discs in which clones homozygous for the wildtype Ubx exon 1 replacement allele were induced. Clones are GFP-negative and marked with a yellow arrow. Cropped images of single clones (outlined) are shown. (E) GFPUbx native fluorescence, and dsRed and Ubx immunostains in haltere discs in which clones homozygous for the GFPUbx fusion allele were induced. Clones are dsRed-negative and marked with a yellow arrow. Cropped images of a single clone (outlined) are shown. (F) GFP native fluorescence, and dsRed and Ubx immunostains in haltere discs in which clones homozygous for the GFP-t2a-Ubx allele were induced. Clones are dsRed-negative and marked with a yellow arrow. Cropped images of a single clone (outlined) are shown. (G) An anti-Ubx immunoblot on protein derived from the following genotypes: yw (lane 1), GFP-Ubx heterozygous (lanes 2, 3), and GFP-t2a-Ubx (lane 4). The lower band is Ubx and the [file pgen.1008444.s008.tif]
