# Supplementary material for: Low affinity binding sites in an activating CRM mediate negative autoregulation of the Drosophila Hox gene Ultrabithorax
Source: PLoS Genet. 2019 Oct 7;15(10):e1008444. doi: 10.1371/journal.pgen.1008444 (PMC6797233; doi:10.1371/journal.pgen.1008444)
Supplement: Attachment — Submitted filename: REVISION_REVIEWERRESPONSE_FINALv2.docx [file pgen.1008444.s009.docx]

Reviewers Commentary in Blue, Author Response in Black

**Point-by-point response to Reviewer #1**

ON/OFF: The distinction of ON versus OFF, i.e., binary expression, is not novel and detracts a bit from the intro. It feels like a “straw man” argument.

We did not intend for this to be a “straw man” argument, rather we wanted to distinguish between two types of transcriptional control that are important for Ubx, our gene of focus. This is particularly important because other published findings regarding Ubx autoregulation find that transient overexpression can result in the complete shutdown of Ubx transcription (a shift from ON to OFF). Here, we have uncovered an autoregulatory mechanism that tunes transcription levels rather than alters transcription state.

Throughout, Red/Green figures should be changed for colorblind readers

This has been changed. Thank you for bringing it to our attention.

Figure 4: Could the authors show the predicted affinity changes from their mutations?

Yes. We have added this to Figure 4 and included it in the text. Because these are clusters of binding sites, the highest relative affinities (MAX relative affinity) for wildtype and mutants are reported.

Figure 4, 5, 6, etc.: “hi” should be change to “high.”

We apologize for the confusion here. We were attempting to use “hi” and “high” to mean two different things. “hi” denotes that a binding site is mutated to a higher affinity and “high” denotes that the wildtype binding site is of a higher affinity than our lower affinity binding sites. We have changed the classification of “high” and “low” to be “Set1 (High Affinity)” and “Set2 (Low Affinity)” to remove this confusion. Now, Hi denotes when a binding site is mutated to a high affinity and Kill denotes when it is mutated to a lower affinity. Now, it should be clear that Set1 and Set2 binding sites are being mutated to lower affinities (Set1^Kill^, Set2^Kill^).

Note: Some figure panels are lower resolution: Fig. 1A; Fig. 3A; Fig. 4S1B.

Thank you for pointing this out. We suspect it occurred due to the compression of files for uploading. We will make sure this doesn’t happen in the final draft.

Fig 6: These are great experiments; it could benefit readers if a different lookup table (LUT) were used (“Fire”). For example, in panel C, the low affinity Mut looks similar to the Cluster2Kill.

We have changed the images in panel C to “FIRE.” Thanks for the suggestion.

Did the authors consider experiments to test how robust the modified endogenous locus is? For, example growing at elevated temperatures (Frankel et al., 2010; Crocker et al., 2015)? Is there a subtle phenotype (Tsai et al., 2019)? For example, smFISH could be compared to Ubx protein to look at noisy expression (or precision). Was there any morphological phenotype? These seems like a fantastic genetic reagent, it would be great to see if there are any subtle changes.

We briefly looked into this by growing flies homozygous for endogenous abx mutations at 18C and 30C. We did not detect any differences in Ubx protein levels (by immunostaining), but have not included this data in the paper. We agree that these lines will be great genetic tools for future studies to test a larger set of environmental conditions and/or genetic perturbations. Certainly, the suggestion to combine these studies with smFISH will be useful in these future studies, but we believe that it goes beyond the scope of this paper.

Discussion: have the authors considered how “hubs” may buffer the native locus? It would be interesting to consider local repression hubs across this region. Such a mechanism could function to stabilize the gene-expression output even if some low affinity binding sites are deleted.

Yes, we have definitely considered the recruitment of abx to repressive hubs as a mechanism for how autoregulation occurs. Directly asking this question will be the topic of future studies. As for this paper, we agree that recruitment to hubs even in the presence of mutated Ubx binding sites could provide robustness to the system, thus masking any effect on Ubx expression levels. We have added this possibility in the discussion.

**Point-by-point response to Reviewer #2**

However, the paper does have several weaknesses. First, while I agree with the authors that we know a more about the regulation of gene expression at the level of ON vs OFF and less about how appropriate levels are achieved – it is unclear if the difference in Ubx expression levels between proximal and distal regions of the haltere disc has any physiological consequence. Because manipulation of the endogenous abx locus failed to disrupt proximal-distal Ubx expression bias, important questions like the following cannot be addressed: Is the differential proximal-distal Ubx expression required for haltere development, and would de-repression of Ubx in the proximal compartment of the haltere disc result in a phenotype/defect? Hence, the biological relevance of studying Ubx mediated autoregulation in the proximal haltere disc has not be established. In addition, I have several other concerns/suggestions, most of which are relatively minor, in regards to the manuscript.

We agree that it would be great to provide a physiological consequence of perturbing proximal Ubx levels. Unfortunately, this is a challenging experiment to do because, despite several attempts, it has been challenging to produce viable adults with modestly higher expression of Ubx protein in the proximal compartment. However, although we cannot yet provide functional data, we know from published butterfly studies that the proximal-distal bias of Ubx is evolutionarily conserved in the wing primordia, suggesting a conserved function. In response to this comment, we now mention this in the revised Discussion.

1) Taking the abx enhancer out of its locus and studying it as a transgene permitted the detailed analysis for the requirement of specific binding sites – revealing that mutating the low affinity Ubx/Exd sites flattened the P/D differences in levels and increasing the affinity of sites increased the P/D levels. Based on these findings and clonal mutant analysis for Ubx, Hth, and Exd, the authors argue that Ubx requires low affinity sites to establish the differences in P/D expression. However, from the pictures in Fig 3C – it appears that the wing disc shows a similar P to D ratio in expression and this imaginal disc does not express Ubx. Does this mean that additional Hox factors (such as Antp) can regulate P/D expression in the wing or is this a Hox/Exd independent regulation? Is Hth and/or Exd required for this P/D difference in the wing as well? Answering this question could reveal new insight into Hox specificity or that there is also a Hox independent mechanism that can impact differences in P/D levels.

The reviewer is correct in noticing that the PD bias of *abx-lacZ* transgenes also exists in the wing disc, which is interesting given the absence of Ubx. We have not directly tested whether Antp+Exd can perform this repressive role, but given the proximally-restricted expression of Antp in the wing this is our preferred model. We expect that PD bias in the wing would also be dependent on Hth and Exd, which are also present in the proximal compartment. However, because the *abx* LacZ transgene shows ectopic activity in the wing, inconsistent with Ubx expression itself (and likely due to the absence of Polycomb inputs in this reporter), we consider the wing activity an artifact of the minimization of the *abx* region. Thus, we have been hesitant to use it as a tool to study regulation within the wing. With that said, we have mentioned the possibility of Antp-mediated proximal repression in the text referring to Figure 3.

2) The authors provide evidence based on the LacZ transgene that low affinity sites are required for a proper P/D ratio of expression as raising the affinity steepens the differences seen. But if this low affinity site is required for proper P/D ratios, shouldn’t changing the low affinity sites to high affinity sites also impact the P/D ratios when inserted into the endogenous locus? In other words, while I understand and agree with the authors argument that the lack in change of P/D ratios when the endogenous sites were mutated could be due to redundancy via other elements with low affinity sites, I would have predicted that changing the low affinity sites to high affinity would have been dominant – and now one would need lower levels of Ubx to shut itself off and thus a steeper P/D ratio should be observed. The fact that no significant difference is observed calls into question whether the Ubx/Exd site just happens to be low affinity versus the Ubx/Exd site must be low affinity. This also ties into my above argument that unfortunately we do not know if the differences in P/D Ubx expression levels have a physiological consequence. Hence how important the sites must be low affinity is unclear.

We also expected that the mutation of an endogenous binding site to higher affinity, which as the reviewer points out is a gain-of-function experiment, would be more likely to affect Ubx expression levels than abrogating the binding site. Thus, we were similarly surprised to see the result. However, the data suggest that even in this gain-of-function scenario, the system must be sufficiently buffered to prevent a change in expression levels at the endogenous locus. Note that in these experiments we are only changing a single binding site cluster to high affinity and it appears that this does not significantly alter the combined affinity of all binding sites in the region, and thus the recruitment of Ubx to the locus. The generation of alleles with additional high affinity binding sites in the future should be able to better address this question. We now discuss these ideas in the Discussion. Further, as this manipulation failed to alter Ubx levels, these data are not relevant to the question of whether Ubx down-regulation in the proximal compartment is important for haltere development. In fact, based on the observation that the system is so well buffered, one could argue that the down-regulation is particularly important.

3) Ubx only seems to negatively autoregulate its expression in the proximal compartment, and the same sites in the abxN element are repressed by Ubx proximally but not distally. Is the high-proximal low-distal expression of Hth and nuclear localization of Exd shown in figure 2 responsible? While it is clear these factors are required for proximal repression, would overexpression of Hth in the distal compartment using the nub-Gal4 presented in figure 7, result in distal repression?

From our data, it does seem that Ubx only negatively autoregulates in the proximal compartment; and, based on our genetic experiments (null clones of Hth and Exd, and GFP-degrade of Ubx) this effect is dependent on the activity of Ubx along with Hth and Exd. We have conducted experiments where we express Hth distally and tissue fate is severely altered. Images of adult halteres in which we have expressed Hth^HM^ throughout the haltere beginning in early larval development (and continuing until adulthood) show the loss of the distal portion of the haltere (the capitellum). Thus, the state of these cells has very clearly changed in response to the overexpression of Hth, making it difficult to interpret whether an effect on Ubx is due to Hth alone or the indirect effects of changing the cellular state.

There are data in the paper that suggest that the activity of Ubx (in complex with Hth and Exd) differs in different spatial contexts. For example, the distal hinge region, which is Hth+, also exhibits high Ubx expression. This is in contrast to repression in the proximal domain. Thus, the presence of Hth/Ubx/Exd is not always sufficient for autoregulation. We do not yet have a mechanism to explain this, but it is brought up in the Discussion. Further, we do not yet know if Ubx monomer (in the absence of Hth/Exd) is bound to *abx* and acts in a non-repressive manner. These are all interesting questions for further investigation.

4) Figure 4 shows that mutations to the predicted Ubx-Exd site in cluster 1 of abxN change proximal-distal expression bias decreasing the Distal to Proximal ratio when mutated to kill binding and increasing the ratio when the site is strengthened. However, it is less clear based on the images in 4C if this observation is due to changes in proximal expression levels (de-repression with Cluster1-kill and increased repression with Cluster1-high) or due to changes in distal expression levels. From the images shown it seems that distal expression levels are decreased in Cluster1-kill compared to WT which would decrease the D/P ratio, but would not support the hypothesis that the sites are required for repression in the proximal compartment. Could quantified proximal and distal levels be shown to indicate that not only does the D/P ratio change, but it is the result of de-repression/increased repression in the in the proximal haltere disc.

Yes, as pointed out in the text, the data indeed show that the levels of the *abxN* reporter gene change in both in the distal compartment and the proximal compartment, both for the kill and Hi affinity mutations (Figure 4C). However the decrease in distal levels is not observed when Ubx binding sites are mutated in the larger *abxF-lacZ* reporter gene, but proximal levels are still derepressed in this context (Figure 6C). Thus, the increase in distal levels that the reviewer noticed for the *abxN-lacZ* transgene appears to not hold up when a larger fragment is analyzed.

When we quantify the “raw” intensities for both the proximal and distal compartments, the trends are the same (proximal levels increase for both *abxN* and *abxF*, distal levels decrease only for *abxN*) but the disc-to-disc and experiment-to-experiment variation for these values is too high to quantify these trends, which is why we focused on showing the internally controlled proximal:distal ratios. However, to show the trends in the raw values we now include an example obtained for one experiment in Supplemental Figure S4D.

5) Minor: In Fig 4C – I believe the Y-axis should be labeled “Distal/Proximal bgal levels” and not “Distal/Proximal Ubx levels”.

Yes. Thank you. This has been changed.

6) Minor: I may have missed this and if I did the authors can ignore this comment, but is homozygous removal of the abx enhancer lethal? I assume since they are only showing clonal analysis, that it probably is, but it would be nice to state. Moreover, do any of the clonal enhancer mutants develop into adults and do they show haltere to wing transformations? Also, do the “patchy: AxbF and AxbN rescue lines” show haltere to wing transformations?

Deletion of abxFAIRE only rarely results in adults with the homozygous mutation. These flies exhibit a moderate haltere-to-wing transformation, but they are so rare that we cannot study them thoroughly. We have not allowed any of the clonal enhancer mutants to develop into adults; however, we would not expect that they have a phenotype because homozygous enhancer mutants do not. The patchy *abxN* rescue lines also show slight haltere to wing transformation that are variable in their severity. This information has been added to the Materials and Methods section.

**Point-by-point response to Reviewer #3**

The authors convincingly show that Ubx, acting with Exd and Hth, negatively autoregulates. They also show that they can perturb this regulation in a minimal enhancer reporter. However, they did not identify DNA sequences whose mutation changes expression in the endogenous locus. The experiments are well-done, but ultimately, the story is incomplete. For publication in Plos Genetics, the authors would need to provide some insight into the negative feedback mechanism. For example, one simple hypothesis is that a shadow enhancer compensates for perturbation to abxF. If the authors could provide evidence for a shadow enhancer, this would greatly strengthen the paper. Alternatively, the authors could provide mechanistic insights with other types of experiments of their design.

We would of course love to understand the mechanism of how Ubx expression levels are buffered against *abx* mutations in the endogenous locus in greater detail than the present study permits. This was the motivation behind increasing the size of DNA sequence we examined at the endogenous locus and the number of binding sites that we mutated; if the solution was the presence of an easily defined shadow enhancer, we would have found it. However, given the large number of CRMs and predicted binding sites within the unusually large *Ubx* locus, in addition to the large number of accessible regions, it may not be possible to narrow this effect down to a small number of well-defined shadow enhancers. In fact, this is one of our conclusions; that understanding gene regulation at the endogenous *Ubx* locus will require considering the locus as a whole, as opposed to focusing on discrete regulatory elements. We spend time in the Discussion making these points more clearly.

Finally, the writing of the manuscript is dense and cryptic. The authors should edit the paper rigorously to make it more reader friendly. I list some of the passages that were extremely challenging to decipher:

“Importantly, we also reveal that Ubx-Exd-binding site mutations sufficient to result in de-repression of abx activity in the proximal haltere in a transgenic context are not sufficient to de-repress Ubx expression when mutated at the endogenous locus, suggesting the presence of multiple mechanisms through which Ubx-mediated repression occurs.”

“Though often described as the blueprint of an organism, the genome – and its underlying code – remains undecipherable unless understood within a cellular context. This is because, in addition to genomic sequence, knowledge of the subset of genes expressed throughout space and time is required.”

“While this demonstrates the importance of an ON/OFF binary mode of control for Ubx expression during development, it is also important to note that, although Ubx is detected in all cells of the haltere disc, distinct levels of Ubx expression occur along the proximal distal axis.”

“This observation raises questions not only of the downstream effects of high versus low Ubx expression on cell- and tissue-fate, but also of the mechanisms by which Ubx levels are regulated in proximal versus distal compartments. While the first question remains unanswered, here, we address the latter question and provide evidence for the presence of an autoregulatory mechanism in which Ubx directly represses its own expression within the proximal compartment of the developing haltere.”

Although it is not always clear to us why the reviewer finds these passages difficult to decipher, we have done our best to revise the manuscript to make it readily accessible to all biologists.

**‘not shown’**

In our initial submission we used the phrase ‘not shown’ to indicate that the graph in panel 6A does not show **all** binding sites; only the top 20 highest affinity binding sites are represented in the graph. The term ‘not shown’ did not refer to data not being shown, just the representation of predicted sites in the graph to ones that have an affinity above non-specific binding. In the revision, we changed the terminology to “not represented in figure” to more accurately represent what we meant.
